# Therapeutic Implications of Diet in Inflammatory Bowel Disease and Related Immune-Mediated Inflammatory Diseases

**DOI:** 10.3390/nu13030890

**Published:** 2021-03-10

**Authors:** Yan Jiang, Karolin Jarr, Cosima Layton, Christopher D. Gardner, Judith F. Ashouri, Maria T. Abreu, Sidhartha R. Sinha

**Affiliations:** 1 Division of Gastroenterology & Hepatology, Department of Medicine, Stanford University, 300 Pasteur Drive, M211, Stanford, CA 94305, USA; yjiang24@stanford.edu (Y.J.); jarr@stanford.edu (K.J.); 2 University of North Carolina at Chapel Hill, Chapel Hill, NC 27599, USA; cosi@live.unc.edu; 3 Stanford Prevention Research Center, Stanford University School of Medicine, Stanford, CA 94305, USA; cgardner@stanford.edu; 4 Division of Rheumatology, Department of Medicine, University of California, San Francisco, CA 94143, USA; Judith.Ashouri@ucsf.edu; 5 Division of Gastroenterology, Department of Medicine, Leonard Miller School of Medicine, University of Miami, Miami, FL 33136, USA; mabreu1@med.miami.edu

**Keywords:** inflammatory bowel diseases, ulcerative colitis, Crohn’s disease, diet

## Abstract

Despite being a focal issue to patients, the effect of diet on adult inflammatory bowel disease (IBD) remains underexplored with limited guidance. While promising clinical trials are currently underway, there is a need for further evidence-based recommendations. As such, we summarize the current evidence on various diets used in the treatment of IBD and also explore the potential applications of dietary data from related immune-mediated inflammatory diseases (IMIDs), such as rheumatoid arthritis and psoriasis, to provide additional information to inform IBD providers. To date, there have been multiple diets investigated as adjunctive therapy in IBD, but many associated studies are small, non-randomized, and not controlled. Mediterranean, vegetarian/vegan, and reduced-calorie/fasting diets have been studied and have shown some positive results in other IMIDs, which may suggest potential applicability to those with IBD, but larger, well-designed clinical trials are needed for further guidance. Gluten-free and low fermentable oligosaccharides, disaccharides, monosaccharides, and polyols fermentable oligosaccharides, disaccharides, monosaccharides, and polyols (FODMAP)diets do not appear to have an impact on IBD disease activity, but low FODMAP may potentially be helpful for those with concurrent functional gastrointestinal symptoms. Specific carbohydrate diets have been mainly assessed in children but show some potential in small adult studies.

## 1. Introduction

Inflammatory bowel disease (IBD), which includes Crohn’s disease (CD) and ulcerative colitis (UC), is characterized by chronic inflammation of the gastrointestinal tract and affects about 1.6 million adults in the United States [1]. Given limited evidence on non-pharmacologic therapies and concerns around immunosuppressive therapy, the role of diet in managing the disease is one of the most common questions patients with IBD have [2]. Prior studies have demonstrated that one’s diet may have an impact on the risk of developing IBD [2]. These have shown that those who eat more fruits and vegetables may have a lower risk and those who consume more animal fat and sugar may have increased risk [3,4,5,6,7]. Though epidemiological data suggest certain dietary factors may influence the development of IBD, it remains unclear which foods may influence disease progression and flares [2]. Various components of diets have been suggested as potential exacerbating anti-nutritive factors, including processed foods and their additives [8]. Low vitamin D levels have also been associated with increased inflammation, and other supplements, such as curcumin, may have anti-inflammatory properties as well [9]. Over half of patients with IBD believe that their dietary habits could trigger flare-ups [10]. Yet, due to limited knowledge, there remains no clear dietary guidance for adults with IBD [11]. The majority of patients report receiving inadequate guidance from providers [12]. 

**Figure 1:**
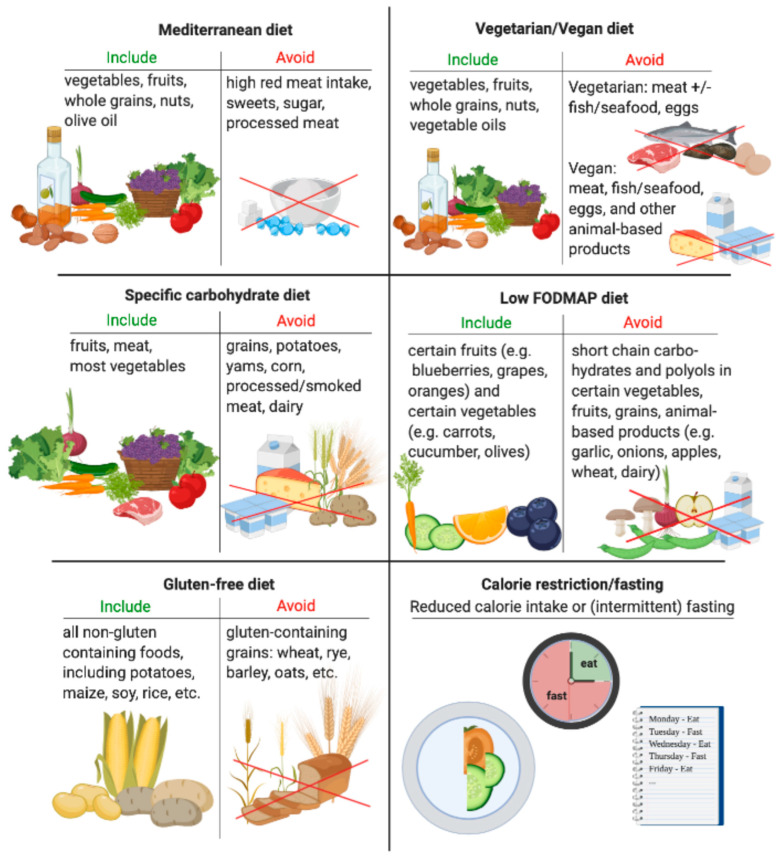
Components of common diets studied in irritable bowel disease (IBD).

Though mostly studied in the pediatric and adolescent population, enteral nutrition has been shown to improve outcomes and inflammation in those with CD [13,14]. The efficacy and tolerability in adults, however, are less clear, with a meta-analysis concluding that there is limited evidence for the potential benefit of elemental diets in the maintenance of remission in adults with CD [2,15]. Although not the focus of our review, an impetus for the development of certain restriction diets was based on the success of such enteral nutrition studies. To date, there have been multiple diets (Figure 1), including the Mediterranean, specific carbohydrate diet (SCD), and low fermentable oligosaccharides, disaccharides, monosaccharides, and polyols as indicated (FODMAP)studied in patients with IBD, but these studies are quite heterogeneous in design and levels of supporting evidence [11]. While additional promising dietary studies in IBD are underway, there is a need to find further evidence-based recommendations [16,17,18,19]. Understanding the role of diet in other immune-mediated inflammatory diseases (IMIDs) offers an opportunity to provide additional dietary guidance to patients with IBD.

There have been several recent reviews of dietary interventions in IBD [2,12,20,21]. In this review, we not only summarize the evidence supporting the role of various diets in the treatment of adult IBD but also—due to the limited data supporting actionable interventions—explore the role of diet in the management of other chronic inflammatory conditions, namely rheumatoid arthritis (RA) and psoriasis/psoriatic arthritis. The rationale here lies in the fact that there are several areas of overlap between IBD and these IMIDs. These disorders are often characterized by frequently debilitating inflammation and share common treatment regimens reliant on targeting overlapping biological pathways [22,23]. Many patients with IBD also have arthropathy or psoriasis, and these diseases are not addressed by newer agents, such as gut-specific anti-inflammatories [24,25,26]. Hence, analyzing the RA and psoriasis literature also has the added potential benefit of seeing which dietary modifications may improve these frequently coexistent conditions.

## 2. Materials and Methods

We performed a literature review using the PubMed database to identify relevant articles related to diet as adjunctive therapy in IBD and other IMIDs. We searched using terms commonly used to describe the IMID (e.g., ulcerative colitis, Crohn’s disease, rheumatoid arthritis, psoriasis, etc.) combined with terms to describe the diet of interest (e.g., Mediterranean diet, low FODMAP, gluten-free, etc.). We reviewed a variety of types of research articles, including systematic reviews and meta-analyses, randomized controlled trials, and observational studies. We generally limited our data to adult studies; however, in areas where clinical research was limited in the area (e.g., specific carbohydrate diet), some pediatric studies were included as well. Though not a systematic review, studies were excluded if they were deemed lower quality (very small sample sizes, poor methodology) by a consensus among our research team, which included gastroenterology and rheumatology physicians.

## 3. Results

### 3.1. Mediterranean Diet

#### 3.1.1. IBD

The Mediterranean diet (MD) is typically high in vegetables, fruits, whole grains, nuts, monounsaturated fats, such as olive oil, and low in red meat. Initial interest in this diet began with observations of lower rates of cardiovascular disease mortality in the Mediterranean region compared to the United States [27,28]. Indeed, subsequent studies have shown an expanded list of long-term benefits of this diet, including weight loss and reduction in C-reactive protein (CRP) [29]. The data around MD use in the IBD population, however, are currently limited. A Greek observational study of 86 patients with CD (41 active, 45 in remission) assessed adherence to an MD and its association with disease activity and quality of life [30]. Using a previously studied scoring method termed MedDiet (higher score values indicate higher adherence), the diet of patients in the 6 months preceding the study was assessed. The investigators found that those in remission had higher MedDiet scores than those with active disease (30.2 ± 5.8 vs. 26.8 ± 5.0, *p* = 0.005) [30]. MD adherence was positively correlated with IBD questionnaire (IBDQ) scores (a validated quality-of-life instrument for patients with IBD) and negatively correlated with CRP values and the clinical Harvey–Bradshaw index (HBI), which is used to assess disease severity. A recent analysis of patients with IBD in three large cohorts (the Nurses’ Health Study (NHS), NHS II, and Health Professionals Follow up Study) demonstrated an association with MD adherence and decreased mortality following IBD diagnosis (HR 0.69; 95% CI 0.49–0.98) [31]. In a small study of eight patients with CD who received the MD for 6 weeks, there was a trend toward normalization of the microbiota associated with gut health, but no significant change in post-diet CRP [32]. A large cohort study that was recently published suggested that greater adherence to an MD was also associated with a lower risk of developing CD, but not UC [33]. A recently published study demonstrated slightly lower but not significantly decreased rates of relapse from the remission of CD in those who consumed less meat [34].

#### 3.1.2. RA and Psoriasis

While the data supporting the MD in IBD have been limited, with more studies ongoing, this diet has been widely studied in RA (Table 1). A study of 130 female patients with RA demonstrated that those who received weekly sessions on healthy eating with an MD focus (including hands-on cooking classes) for 6 weeks had significant improvement in RA symptom outcomes and disease activity scores compared to controls who received only written dietary information on general healthy eating [35]. Notably, there was no change in CRP in the intervention group. A recent large Swedish-based population study found that higher adherence to an MD was associated with a lower odds ratio of developing seropositive RA [36]. Interestingly, when controlling for gender, this effect was only seen in men (OR 0.49; 95% CI 0.33–0.73). This potentially beneficial trend is also seen in patients with psoriasis (Table 1), with several studies showing an inverse relationship between MD adherence scores and disease activity, including a large French questionnaire-based cohort study of over 35,000 patients [37,38]. These collective findings suggest adherence to an MD diet may reduce clinical disease activity in chronic inflammatory diseases, as well as potentially prevent disease onset in a subset of individuals. These data support the hypothesis that an MD will have a positive impact on patients with IBD that warrants further investigation. Indeed, there is a multicenter trial underway that aims to evaluate the effectiveness of an MD (as well as SCD) as adjunctive therapy in patients with CD [18].

### 3.2. Vegetarian and Vegan Diet

#### 3.2.1. RA and Psoriasis

Vegetarian diets vary considerably but generally eliminate meat products, while vegan diets are void of all animal-based food products, including eggs, dairy, and seafood. These diets gained popularity as a potential therapeutic tool for inflammatory diseases after multiple clinical trials with RA patients demonstrated positive results (Table 2). A 1991 clinical study compared a yearlong vegetarian diet vs. control (consumption of ordinary mixed food defined by the patient) in 53 patients with RA [39]. By the end of the year, the 16 patients (of 27 originally randomized to the intervention group) still adherent to the diet experienced significant improvement in their inflammatory markers, erythrocyte sedimentation rate (ESR), and CRP. There was, however, a significant drop-out from the study (34 patients completed the study), and analysis was not performed using intention to treat, limiting the conclusions from this study. A follow-up investigation, however, found that the patients who showed improvement in the initial trial continued to experience improvement two years later [40]. An additional study, following 24 patients with RA, found that a low-fat vegan diet decreased not only patients’ pain scores (*p* < 0.004) but also the number of tender (*p* < 0.01) and swollen joints (*p* < 0.02); these results were also associated with a downward CRP trend [41]. 

Similarly, a study investigating the effects of a gluten-free vegan diet compared to a well-balanced non-vegan diet in 66 patients with RA found a significantly higher percentage of patients in the vegan group fulfilled the American College of Rheumatology (ACR) 20 improvement criteria (defined as an improvement in at least 20% in five of seven RA outcome measures, including tender and swollen joint counts) (40% vs. 4%). For vegan diet responders, CRP also improved significantly [42]. There are limited clinical data regarding the effect of a vegan/vegetarian diet in patients with psoriasis (Table 2), but an observational survey-based study conducted by the National Psoriasis Foundation found that patients generally reported a positive response to a vegan/vegetarian diet and believed it helped their symptoms [43].

#### 3.2.2. IBD

In contrast to the studies in RA, data on vegetarian or vegan diets in IBD are not as robust. However, a cohort study of 191 patients with UC in remission demonstrated an increased association between meat intake and relapse (OR 3.2 (95% CI 1.3–7.8)) [44]. In 2010, one small prospective Japanese study investigated the protective effects of a semi-vegetarian diet (SVD, defined by fish once per week and other animal meat products every two weeks) in patients with CD who recently achieved remission [45]. The study included 22 patients over a two-year period and compared 16 subjects on the SVD diet versus 6 subjects who kept regular omnivorous diets. Maintenance medication profiles were similar, with most receiving either sulfasalazine or mesalamine. Remission was maintained at two years in 15 of 16 subjects in the SVD group compared to 2 of 6 subjects in the omnivorous group (*p* = 0.0003). The omnivorous group in this study was defined as patients who did not follow the SVD regimen (e.g., had more than the defined amount of fish and meat products that were allotted). 

Overall, the RA patient data suggest that a vegetarian/vegan diet could be a potentially promising therapy tool for inflammatory disorders, such as IBD, but it should be noted that these studies do have important limitations given study designs and findings that may have been confounded by other intervention components (such as gluten vs. gluten-free) and limited by high drop-out rates.

### 3.3. Gluten-Free Diet

Gluten is a protein found in many grains, including wheat, rye, and barley, that can activate an aberrant immune response in some individuals. Gluten-free diets are typically used by patients with celiac disease or non-celiac gluten intolerance. This diet has been studied to a limited extent in those with IBD, but additional information can be gathered from the RA and psoriasis patient populations (Table 2). 

#### 3.3.1. IBD

A cross-sectional questionnaire study of 1647 patients participating in the Crohn’s and Colitis Foundation of America (CCFA) Partners cohort aimed to evaluate the prevalence of gluten-free diets in the IBD population [46]. They found that 19% had tried a gluten-free diet, and 8% reported current use. Notably, celiac disease had a 0.6% prevalence in the study participants. Overall, 66% of participants who had tried the diet reported an improvement in at least one of the clinical symptoms related to IBD (bloating, diarrhea, abdominal pain, fatigue, and nausea), and 38% of participants reported fewer or less severe flares while on the diet. A 2017 Swiss IBD prospective cohort study identified nearly 5% of their 1254 patients on a gluten-free diet. In contrast, they did not find gluten-free diets to be associated with IBD activity, hospitalization, or surgery rates [47].

#### 3.3.2. RA and Psoriasis

As previously mentioned, a gluten-free, vegan diet has been reported to improve RA symptoms [39,42]. To our knowledge, there are no other published studies of gluten-free diets alone in patients with RA. In patients with psoriasis, a gluten-free diet led to an improvement in Psoriasis Area and Severity Index (PASI—an index that combines severity and area affected into a single score) in those with elevated gliadin antibodies. It is important to note that while a celiac diagnosis is not mentioned, duodenal biopsy findings were included, and 15 of 33 patients had an increase in the number of infiltrating lymphocytes, and two were found to have villous atrophy—criteria used in the diagnosis of celiac disease. Normal histology was seen in 16 gliadin-positive patients, and PASI was improved in this group as well [48]. This improvement was not, however, observed in the small group without gliadin antibodies. Notably, when patients resumed a regular diet after 3 months of the gluten-free diet, 60% of the patients with elevated gliadin levels required an increase in psoriasis treatment. Interestingly, a higher prevalence of celiac disease in those with psoriasis has been reported in an Italian multicenter study [49]. Given this benefit was seen in a specific psoriasis subpopulation, it is difficult to extrapolate the effect of a gluten-free diet on altering IBD disease activity, especially with the lack of quality IBD data. 

### 3.4. Calorie Restriction/Fasting

#### 3.4.1. IBD

Diets that restrict total calories or include periods of fasting have gained attention as a potential treatment for IMIDs based on animal and clinical studies showing anti-inflammatory effects [50,51]. These studies have largely focused on this type of diet as a treatment for RA and psoriasis (Table 3). A study conducted in 2008, however, analyzed the effects of Ramadan fasting on 60 IBD patients in remission [52]. No adverse effects of fasting on the patients were noted. Additionally, a significant decrease in a clinical colitis activity index (comprising frequency of bowel movements, urgency, blood in the stool, general well-being, and extracolonic features) was noted in patients with UC (2.97 to 1.88, *p* = 0.005). A non-significant trend for improvement in the frequently used metric to evaluate CD severity, the Crohn’s Disease Activity Index (CDAI) in those with CD.

#### 3.4.2. RA and Psoriasis

Low-calorie diets have been studied more extensively in patients with psoriasis. Weight gain in patients with psoriasis has been associated with worsening disease severity [53,54]. A trial evaluating a low-calorie diet for 16 weeks in overweight and obese patients (BMI 27–40) with psoriasis showed improvement in the quality of life measures (*p* = 0.02) and borderline improvement in PASI (*p* = 0.06) when compared to control [54]. It is important to point out that weight loss was 15.4 kg greater in the intervention group (*p* < 0.001). Years later, the same group published longer-term data on a subgroup of patients from the original cohort. The data suggest that those in the intervention group had sustained reduction in PASI at 64 weeks (mean −2.9; 95% CI: −3.9, −1.9) [55]. A more recent Swedish study evaluated a low-calorie diet and its impact on psoriatic arthritis disease activity. Notably, there was no control group, but those who received the diet lost a median weight of 18.7 kg and showed improvements in multiple disease activity parameters: tender joint count, CRP, and measures of global health, pain, and fatigue [56].

Though there have been studies demonstrating the benefit of initial short spans of fasting combined with various diets, such as vegetarian, data strictly on fasting remains limited in RA patients [39,40]. Two small German studies of patients with RA (some patients with fibromyalgia were also included) undergoing about a week of modified fasting periods (protocol including 8 days with a 300-kcal daily limit) reported a small reduction in RA disease activity scores [57,58]. There is also an ongoing clinical trial in Europe examining the effectiveness of therapeutic fasting (time-restricted eating hours) on patients with RA [59].

Based on initial evidence and data from other chronic inflammatory diseases showing some effectiveness of fasting and/or caloric restriction in the reduction in inflammatory markers and symptoms, further studies in patients with IBD are warranted. Currently, there are two ongoing trials investigating the role of intermittent reduced-calorie diets in IBD [16,17].

### 3.5. Specific Carbohydrate Diet

#### IBD

The specific carbohydrate diet (SCD) is a grain-free diet low in certain carbohydrates. Proponents of this diet hypothesize that complex carbohydrates are difficult to digest and may lead to dysbiosis and inflammation. Clinical data collected on the effectiveness of the diet, however, are limited and are focused on patients with IBD and not other IMIDs (Table 4). In IBD, SCD has been studied primarily in the pediatric population. SCD appears to have a significantly positive impact on Pediatric Crohn’s Disease Activity Index (PCDAI) and Pediatric Ulcerative Colitis Activity Index (PUCAI) scores. In this population, the diet also resulted in a significant improvement in CRP (*p* = 0.002) and calprotectin (*p* = 0.006) (a fecal inflammatory marker) levels [60,61].

**Table 4:** Summary of dietary clinical trials in IBD. Current data suggest that lower meat consumption may avoid relapse, and low fermentable oligosaccharides, disaccharides, monosaccharides, and polyols (FODMAP) can provide relief from functional-like gastrointestinal symptoms.

Study	IBD Type	Design	*N*	Results
Mediterranean Diet (MD)				
Papada [30]	CD	Observational	86	Higher adherence with 6-month MD was associated with higher remission rates (*p* = 0.005).
Lo [31]	CD/UC	Prospective cohort study	828	Higher adherence with MD was associated with decreased mortality following IBD diagnosis (HR 0.69; 95% CI 0.49–0.98).
Marlow [32]	CD	Uncontrolled study	8	6-week MD showed trend for normalization of microbiota, no effect on CRP (decrease less than 1 mg/L, *p* = 0.39).
Khalili [33]	CD/UC	Prospective cohort study	83,147	Higher adherence with MD was associated with a lower risk of developing CD (*p* = 0.03), but not UC (*p* = 0.61).
Albenberg [34]	CD	Prospective, controlled cohort study	214	Lower red and processed meat consumption were associated with lower relapse rates (42% vs. 62%) but no difference in time to relapse.
**Vegetarian/Vegan Diet**				
Chiba [45]	CD	Prospective controlled study	22	Lower relapse rate in patients on semi-vegetarian diet (1/16, 6%) vs. omnivorous diet (4/6, 67%) (*p* = 0.0003).
Jowett [44]	UC	Prospective cohort study	191	Higher consumption of meats (OR 3.2; 95% CI 1.3–7.8), particularly red and processed meat (OR 5.19; 95% CI 2.1–12.9), protein (OR 3.00; 95% CI 1.25–7.19), and alcohol (OR 2.71; 95% CI 1.1–6.67) increased the likelihood of relapse.
Amarapurkar [7]	CD/UC	Prospective case-control study	1054	Vegetarian diet was a protective factor for UC (OR 0.29; 95% CI 0.27–0.39) and a risk factor for CD (OR 1.179; 95% CI 0.88–1.57).
**Gluten-Free Diet (GFD)**				
Herfarth [46]	CD/UC	Cross-sectional questionnaire study	1647	66% of participants report an improvement in clinical symptoms when on GFD, although the prevalence of celiac disease was only 0.6%.
Schreiner [47]	CD/UC	Prospective cohort study	1254	GFD was not associated with IBD activity, hospitalization, or surgery rates.
**Calorie Restriction/Fasting**				
Tavakkoli [52]	CD/UC	Prospective cohort study	60	Ramadan fasting significantly improved symptoms (CAI reduction of 1.1) in UC (*p* = 0.005) but not in CD.
**Specific Carbohydrate Diet (SCD)**				
Cohen [60]	CD	Prospective, uncontrolled study	10	Significant improvement in disease activity in pediatric CD (PCDAI reduction of 13.3, *p* = 0.011).
Obih [61]	CD/UC	Retrospective chart review	26	Significant improvement in disease activity (PCDAI reduction 11.4 at 6 months, *p* = 0.03), in CRP (−0.9 mg/dL, *p* = 0.03) and calprotectin (−181 mcg/g, *p* = 0.03) in pediatric CD compared to control. No significant improvements in pediatric UC.
Kakodkar [62]	CD/UC	Case series	50	Patients in disease remission report the SCD to be effective in controlling acute flare symptoms (mean = 91.3%, range = 30% to 100%) and at maintaining remission (mean = 92.1%, range = 53% to 100%).
Suskind [63]	CD/UC	Survey study	417	42% of patients report achieving remission at 6 and 12 months while on the diet. 47% of patients report improvement in abnormal lab values.
**Low FODMAP Diet (LFD)**				
Gearry [64]	CD/UC	Retrospective study	72	Improved symptoms after 3 months of LFD.
Prince [65]	CD/UC	Prospective study	88	Significant improvement in functional-like gastrointestinal symptoms compared to baseline (78% vs. 16% at baseline reporting satisfactory relief, *p* < 0.001).
Pedersen [66]	CD/UC	Controlled open-label study	89	LFD improved IBS symptoms (55 points lower IBS-SSS, *p* = 0.02) and health-related quality of life (SIBDQ 10 points higher, *p* < 0.01) compared to normal diet in IBD in remission.
Cox [67]	CD/UC	Single-blind study	52	LFD improved gut symptoms compared to control (52% reporting adequate relief on LFD vs. 16% on control, *p* = 0.007).
Cox [68]	CD/UC	Double-blinded, controlled, re-challenge study	32	Fructose challenge brought less relief of functional-like gastrointestinal symptoms compared with glucose (62.1% reported relief in the fructan group vs. 89.7 in glucose, *p* = 0.033).

More limited studies are available in the adult population. However, one case series of 50 mostly adult patients surveyed the effects of SCD on IBD symptoms [62]. Survey data were collected from patients with confirmed IBD by chart review, following SCD-living for a mean of 9 months. Nearly 70% of patients self-reported no symptoms while on an SCD. Patients also reported the diet to be effective at both controlling flare symptoms and maintaining remission with a mean effectiveness rating of 91.3% and 92.1%, respectively. Most patients (82%) reported implementing this diet due to fear of long-term consequences of IBD medications. One of the study limitations was that it only included patients in clinical remission, biasing their findings to those who may have perceived benefit from SCD [62].

In a separate 2016 survey study of 417 patients with IBD, including a majority of adults, patients perceived that the SCD helped alleviate clinical symptoms; 42% of patients reported achieving remission at both 6 and 12 months while adhering to the diet [63]. Improvement in abnormal lab values was self-reported by 47% of patients. Taken together, these studies suggest that SCD could be of therapeutic benefit to at least a subset of patients with IBD. SCD, in comparison to MD, is currently being investigated in a trial of patients with CD, as mentioned above [18].

### 3.6. Low FODMAP

#### IBD

The low FODMAP (fermentable oligosaccharides, disaccharides, monosaccharides, and polyols) diet (LFD) restricts the consumption of short-chain carbohydrates and sugar alcohols. These compounds are thought to be poorly absorbed by the small intestine, and avoidance has been used effectively in patients with irritable bowel syndrome (IBS) [69]. Fermentable carbohydrates have been shown to exacerbate functional gastrointestinal symptoms at high doses, even in those with IBD [68]. To date, the effectiveness of the LFD has been studied in patients with IBD, but not in those with other autoimmune conditions, such as psoriasis or RA.

In 2009, an initial retrospective telephone questionnaire study found that 72 IBD patients who had received dietary advice on LFD plan reported a decrease in their symptoms after 3 months, with improvements in pain, bloating, and diarrhea [64]. Half of the patients were deemed “responders” to the diet, with improvement defined as at least 5 points out of a 10-point scale on overall symptoms. A subsequent 88 patient prospective study that evaluated the effects of an LFD on IBD patients mainly in remission found a significant improvement in functional-like gastrointestinal symptoms, including abdominal pain, bloating, flatulence, and urgency [65]. All patients received the diet plan, and, at the end of the study, 78% reported satisfactory relief of symptoms compared to 16% at baseline (*p* < 0.001).

A Danish trial of LFD in patients with IBD in remission (83%) or with mild to moderate disease (17%) enrolled 89 patients over a 6-week period, with 44 patients on the LFD and 45 on a normal diet (ND) [66]. At the end of the 6-week period, the IBD patients on the LFD were found to have significantly lower Irritable Bowel Syndrome Severity Scoring System (IBS-SSS) scores compared to patients on the ND (median 115 vs. median 170, *p* = 0.02). The Short Inflammatory Bowel Disease Questionnaire (SIBDQ) scores, which measures the quality of life in patients with IBD, also statistically significantly improved for LFD patients (median 60) compared to the ND patients (median 50) (*p* < 0.01), but the HBI was not significantly different between the two groups. Similar results were found in a trial of LFD vs. control in 52 patients with quiescent IBD. The LFD group experienced greater relief in gut symptoms, reduction in IBS-SSS but did not show any decreases in markers of inflammation [67].

The results of the studies conducted thus far suggest that LFD may be helpful, especially for overlapping functional symptoms in those with IBD. However, the data on whether or not it impacts IBD disease activity is inconclusive. It should be noted that LFD studies can face difficult challenges, including a high placebo response in prior functional bowel disorder trials [70]. Larger randomized clinical trials with specific aims to evaluate IBD disease activity are needed for effect confirmation.

### 3.7. Other Diets Studied in IBD (e.g., Paleo, Atkins, etc.)

A number of more specific diets have been studied to a limited extent in IMIDs, including Paleo, specific anti-inflammatory regimens, and combination diets. The Paleo diet is a food plan emphasizing lean meat, fish, fruits—foods that could be obtained by hunting and gathering—and avoiding dairy and grains. A small study of 15 patients with active IBD on the “autoimmune protocol diet” (which was described as an extension of the Paleo diet) has shown some promise [71]. The autoimmune diet reduces intake of gluten and refined sugars and has a 6-week staged elimination of grains, dairy, eggs, coffee, alcohol, nuts/seeds, and food additives. This is followed by a 5-week maintenance phase. Though there were no control comparators, patients demonstrated a statistically significant reduction in both partial Mayo score (a disease activity score assessing bowel frequency, rectal bleeding, and physician’s global assessment) for UC and HBI for CD. CRP was unchanged. The Paleo diet was also reported to have benefits in skin response in patients with psoriasis in the aforementioned survey study from the National Psoriasis Foundation [43].

The anti-inflammatory diet (IBD-AID) is a diet derived from SCD and consists of foods such as fish, omega-3 eggs, fruits, vegetables, legume flours, yogurt, kefir, etc. A small case series of patients with IBD (eight CD, three UC) reported benefits of the diet, including reduction in symptoms and disease severity scores. It was reported that these patients were also able to reduce at least one IBD medication as well [72]. It should be noted that out of the 40 eligible patients with IBD, there was a significant drop-out (3 not seen by dietician and 13 lost to follow-up). Though it was noted that 24 had a “good response” to the diet, complete data from only 11 patients were reported in this retrospective chart review.

A recently published study examined the effects of low fat, high fiber on those with either mild UC or in remission [73]. Of the 28 subjects randomized, 17 finished the crossover study in which patients received 4 weeks of a low fat, high fiber diet (10% of calories from fat) and 4 weeks of an “improved American diet”, which was a standard diet with 35–40% of calories from fat and an increased amount of fruits, vegetables, and fiber than a standard diet (diet allocation order was random with a 2-week washout period in between). There was an improvement in SIBDQ from baseline after both diets. However, only after using the low-fat diet segment of the crossover study was there a decrease in markers of inflammation.

## 4. Discussion

Much of the ongoing and future mechanistic/pathophysiology-related research in this area has focused on the influence of the microbiome on the host inflammatory response. Given that microbial dysbiosis is a hallmark of many IMIDs and that diet is one of the most important factors shaping the diversity and composition of enteric flora, diet may have the potential to be a preventive and therapeutic strategy. It is widely accepted that the gut microbiome is a contributor to the development of certain inflammatory diseases. It plays a crucial role, for example, in the maintenance of epithelial barrier integrity and the priming and refinement of the mucosal immune system. In mice, a low fiber diet leads to an irreversible loss of microbial diversity thought to contribute to a dysbiotic, pro-inflammatory state [74]. A diet rich in fiber, however, or in fermented foods was able to increase the bacterial diversity and reduce pro-inflammatory cytokines in healthy humans [75].

The implication of enteric flora in the pathogenesis of IBD and RA is evidenced by the fact that rodents do not develop intestinal inflammation and inflammatory arthritis when raised under germ-free conditions, but the diseases can be triggered by exposure to particular microbes [76,77,78,79,80]. Furthermore, the diversion of the fecal stream can prevent the recurrence of CD in patients post-resection [81]. Impaired intestinal integrity and gut permeability following dysbiosis are also factors contributing to the pathogenesis of psoriasis [82].

The microbiota interacts with the host mainly through bacterial components and microbial metabolites, such as short-chain fatty acids (SCFAs), secondary bile acids, and tryptophan. Recent research has provided a better mechanistic understanding of this, demonstrating that dysbiosis-related secondary bile acid deficiency may lead to inflammation, and supplementation can improve this [83]. Related to the microbiome, nutrigenomics is another important area of ongoing research. Genetics has been shown to influence the absorption or metabolism of certain nutrients, such as vitamin A and D, which play a role in various inflammatory conditions, such as IBD [84,85,86]. Genetic variations may be one of the potential reasons why there is such variability in outcomes related to dietary intake in patients with IBD. Future research in this area offers the hope of a more precise and personalized nutrition plan incorporating the genetic contribution to disease and treatment [84].

Though clearly important to patients [2], the potential of diet in treating inflammatory bowel disease remains underexplored. Some organizations have provided expert opinions based on the best evidence available on the influence of various dietary components, such as fruits, vegetables, and red meat, on patients with IBD, but the evidence level for recommendations is often low due to the dearth of high-quality data [20]. Many studies have small sample sizes, lack a control group, and/or have low retention rates. As such, we examined the literature on dietary interventions in other IMIDs, psoriasis, and rheumatoid arthritis, to provide additional potentially related evidence that could help providers offer more informed dietary guidance.

The Mediterranean diet seems to have promise across IMIDs based on larger cohort and survey-based studies in patients with RA. It has been studied to a lesser extent in IBD with some positive results. Fasting/reduced-calorie diets are also promising interventions. It has been studied in various inflammatory disorders and essentially healthy subjects with benefits shown in many facets of disease as well as common measures of health, such as reduced blood pressure and weight [50,87]. Further studies are warranted to demonstrate the beneficial effect in the IBD population, and care will be needed to select the right subpopulation, as those with severe disease may be significantly malnourished and not ideal candidates for caloric restriction while those who are obese may experience greater benefits [88].

Vegetarian diets appeared, at least in the limited studies identified, to be beneficial in the rheumatoid arthritis patient population. However, some of these studies included potentially confounding elements of other diets, such as fasting. Nonetheless, it does give some evidence that could lead to further evaluation in patients with IBD. The other main diets we evaluated, low FODMAP and gluten-free, did not seem to have a notable impact on IBD disease activity. However, a low FODMAP diet did appear to help symptoms in those with functional gastrointestinal symptoms.

Overall, based on our literature review, Mediterranean diet, plant-based diets, and reduced-calorie diets (in patients with low risk of nutritional deficiencies) may have potential for patients with IBD and other IMIDs and are currently being actively studied. Clearly, the need for additional investigation with well-designed clinical trials will be essential to provide further guidance.

## Figures and Tables

**Table 1:** Summary of the Mediterranean diet (MD) studies in rheumatoid arthritis and psoriasis with potential applicability to irritable bowel disease (IBD). Collective data show adherence to an MD may reduce clinical disease activity in inflammatory diseases. Based on this and growing evidence in IBD, it is reasonable to consider recommending an MD in patients with IBD, but clearly, this warrants further investigation.

Study	Disease	Design	*N*	Results
McKellar [35]	RA	Prospective	130	6-week intervention to MD focused diet showed improvement in patient global assessment (*p* = 0.002), pain scores (*p* = 0.049) and morning stiffness (*p* = 0.041) at 6 months when compared to control.
Johansson [36]	RA	Population case control	5388	Higher adherence to MD was associated with decreased odds in developing seropositive RA (OR 0.79; 95% CI 0.65–0.96).
Phan [37]	Ps *	Population survey	35,735	Higher adherence to MD was associated with lower psoriasis disease activity (OR 0.71; 95% CI 0.55–0.92).
Barrea [38]	Ps	Case control	124	Psoriasis severity scores associated with adherence to MD, *r* = −0.6 (*p* < 0.001).

* Ps = Psoriasis.

**Table 2:** Summary of vegetarian/vegan and gluten-free diet studies in rheumatoid arthritis and psoriasis with potential applicability to IBD.

**Vegetarian/Vegan: Positive Outcomes in RA Studies Suggest Possible Benefits in IBD, Where Data on This Diet Have Been Quite Limited.**				
**Study**	**Disease**	**Design**	***N***	**Results**
Kjeldsen-Kragh [39,40]	RA	Randomized trial	53	Improvement in ESR (−4 mm/h)/CRP (−6 mg/L) (*p* < 0.002 and *p* < 0.005) seen in intervention group. However, significant dropout in study (~60% completed).
McDougall [41]	RA	Single-arm intervention	24	Improvement in RA pain scores (*p* < 0.004), swollen joints (*p* < 0.02) after switch to vegan low fat diet.
Hafstrom [42]	RA	Randomized trial	66	Higher prevalence of fulfilling ACR improvement criteria in those in the vegan diet free of gluten group (40% vs. 4%). 60% intervention group completed the 9-month follow-up.
Afifi [43]	Ps	Survey	1206	Self-reported improvement in skin symptoms in 70% of those on a vegan diet.
**Gluten-free ^+^: ** **Benefit of this diet seen in only a subset of psoriasis patients with gliadin antibodies, which makes it difficult to extrapolate to patients with IBD.**				
**Study**	**Disease**	**Design**	***N***	**Results**
Michaelsson [48]	Ps	Single-arm intervention	39	Gluten-free diet led to an improvement in PASI (5.5 before vs. 3.6 after) in those with gliadin antibodies (*p* = 0.001).

^+^ Kjedsen-Kragh and Hafstrom studies (included in the vegetarian/vegan section above) included gluten-free components of the vegan intervention diet. ESR: erythrocyte sedimentation rate. CRP: C reactive protein. ACR: American College of Rheumatology. PASI: Psoriasis area and severity index.

**Table 3:** Summary of caloric restriction/fasting studies in rheumatoid arthritis and psoriasis with potential applicability to IBD. Data have been promising for both systemic and anti-inflammatory benefits. This diet could be of benefit to specific groups of patients with IBD who are not malnourished.

Study	Disease	Design	*N*	Results
Jensen [54]	Ps	Randomized trial	60	Caloric restriction group showed significant weight loss (−15.4 kg) (*p* < 0.001) and reduction in PASI (−2.0) (*p* = 0.06) compared to regular diet group.
Jensen [55]	Ps	Prospective observational	38	Long-term (>1 year) benefits in both weight loss and PASI for those who underwent a 16-week caloric reduction (PASI reduction mean −2.9; 95% CU −3.9, −1.9).
Klingberg [56]	PsA *	Single-arm intervention	46	Treatment with caloric restriction led to weight loss and significant improvement in multiple symptoms (e.g., VAS pain *p* = 0.004, swollen joints score *p* = 0.021), CRP (−2.0 mg/L) in those with psoriatic arthritis (*p* = 0.041).
Abendroth [57]	RA	Non-randomized Observational	50	Of the 22 who participated in fasting, there were decreased disease activity scores (−1.6) when compared to baseline before dietary intervention at day 13 (*p* < 0.001).
Michalsen [58]	RA	Non-randomized Observational	51	Of the nine patients who fasted, there was a significant improvement in disease activity at 2 weeks compared to baseline (*p* = 0.007).

* PsA = Psoriatic arthritis.

## Data Availability

Not applicable.
